# A boundedly rational model for category learning

**DOI:** 10.3389/fpsyg.2024.1477514

**Published:** 2024-12-09

**Authors:** Troy M. Houser

**Affiliations:** ^1^Department of Psychology, University of Oregon, Eugene, OR, United States; ^2^Institute of Neuroscience, University of Oregon, Eugene, OR, United States

**Keywords:** category learning, autoencoder (AE) neural networks, concept learning, generalization (psychology), RULEX, rate distortion theory, efficient coding theory

## Abstract

The computational modeling of category learning is typically evaluated in terms of the model's accuracy. For a model to accurately infer category membership of stimuli, it has to have sufficient representational precision. Thus, many category learning models infer category representations that guide decision-making and the model's fitness is evaluated by its ability to accurately choose. Substantial decision-making research, however, indicates that noise plays an important role. Specifically, noisy representations are assumed to introduce an element of stochasticity to decision-making. Noise can be minimized at the cost of cognitive resource expenditure. Thus, a more biologically plausible model of category learning should balance representational precision with costs. Here, we tested an autoencoder model that learns categories (the six category structures introduced by Roger Shepard and colleagues) by balancing the minimization of error with minimization of resource usage. By incorporating the goal of reducing category complexity, the currently proposed model biases category decisions toward previously learned central tendencies. We show that this model is still able to account for category learning performance in a traditional category learning benchmark. The currently proposed model additionally makes some novel predictions about category learning that future studies can test empirically. The goal of this paper is to make progress toward development of an ecologically and neurobiologically plausible model of category learning that can guide future studies and theoretical frameworks.

## Introduction

Conceptual knowledge is a defining characteristic of human intelligence. A powerful way that conceptual knowledge is used is by generalizing it to novel situations, enabling efficient and adaptive behavior (Shepard, 1957, 1987, 1994). For example, when we go to a new grocery store, we can generalize previously acquired knowledge about grocery store layouts to infer that the cheese will be close to the milk. A concept is a mental representation of a category (Goldstone et al., 2018). Thus, the concept of a snake refers to the mental representation of a subjectively constructed category labeled *snake*. Given that categories are constructed by individuals to organize their personal experiences, there are numerous possibilities for *how one* might categorize. Despite considerable advancements in the field, there remains a lack of consensus among researchers regarding the psychological nature of categories. In what follows, we introduce a boundedly rational theoretical framework and novel extension of a previously posited process-level computational model that can capture key aspects of human category learning and memory. The guiding notion is that concepts are boundedly rational representations of categories.

### Bounded rationality when acquiring category knowledge

Humans make decisions based on internal representations of external variables (Gershman and Daw, 2017; Niv, 2019), but how such variables are encoded and subsequently decoded to make a decision remains an open question. In real-world decision making, biological systems often have to infer latent states (e.g., categories). Many cognitive models of categorization decisions assume veridical internal representations of categories (Nosofsky, 1986; Nosofsky et al., 1994a,b). Substantial work in reinforcement learning and magnitude discrimination suggests that some amount of noise is inevitable in internal representations (Azeredo da Silveira et al., 2021; Barretto-García et al., 2023; Li et al., 2017; Prat-Carrabin and Woodford, 2022, 2024; Spitzer et al., 2017). This is to say that it is likely infeasible for biological systems to encode and decode information without error. According to the principle of efficient coding (Barlow, 2013), biological systems should seek to maximize representational precision *while minimizing resource consumption*.

The category learning model proposed by Kurtz (2007), called the DIVergent Autoencoding (DIVA), has made important advances in making the modeling of category judgements more biologically realistic. DIVA is a neural network model that utilizes an autoencoder architecture. Autoencoders traditionally learn stimulus mappings in an unsupervised fashion. They have three main components: (1) an encoder, (2) a bottleneck, and (3) a decoder. The encoder takes input data and transforms it to a low dimensional space (the bottleneck). The bottleneck is a form of data compression, or dimensionality reduction, often employed in statistical methods like principal component analysis (PCA) or t-distributed stochastic neighbor embedding (t-SNE). It forces the model to extract out statistical regularities in the data, effectively shedding the irrelevant information, and therefore minimizing resource expenditure. Then these compressed representations are decompressed by the decoder, which transforms them back into their original dimensions, so as to reconstruct the input. Decoding is not trivial, as it is decoding *from the bottleneck*. In other words, the decoder attempts to reconstruct the input after getting rid of some of its original signal, consistent with the notion from efficient coding theory that biological systems have to balance representational precision with resource expenditure. Low reconstruction error indicates that the bottleneck extracted regularities well. Given that an autoencoder's function is to reconstruct the original input, it is typically not an architecture used to model supervised learning, which attempts to make discrete decisions. However, DIVA makes use of a divergent output layer that enables it to make categorical decisions. We discuss this feature below.

However, the traditional autoencoder can have trouble with generalizing because it can overfit to the data (Monshizadeh et al., 2021), by simply reconstructing learned exemplars rather than a category's central tendency (Bozkurt et al., 2021). Reconstructing a category's central tendency should facilitate broader generalization abilities. To circumvent this issue, we use a variational autoencoder (VAE; Kingma and Welling, 2019).

Rather than deterministically mapping inputs to the bottleneck component, VAEs map inputs to probability distributions, thereby adding a stochastic element and enabling generation of diverse outputs. Moreover, rather than sampling directly from these learned distributions [$z\sim N\left(\mu ,\;\sigma^{2}\right)$], which would be computationally intractable, VAEs use the “reparameterization trick” (Kingma et al., 2015). The reparameterization trick expresses the latent probability distributions as deterministic functions of their first two moments: *z* = μ+σ·ε, where ε is noise (which is a random sample from a 0 mean Gaussian with unit variance, see Kingma et al., 2015; Kingma and Welling, 2019). This trick makes the sampling procedure differentiable, which in turn allows the model parameters (μ and σ) to be updated through gradient descent optimization. The loss function that gets optimized is also unique for VAEs. It is a sum of two forms of loss, which is the key theoretical contribution that making DIVA variational makes. The loss function for VAEs is the sum of reconstruction error and the discrepancy between prior and posterior distributions for a sampled latent variable *z*. Reconstruction error is equivalent to distortion in rate distortion theory. It is a measure proportional to the mean squared error between the input and the reconstruction of the input produced by the decoder. The discrepancy between prior and posterior distributions is known as the Kullback-Leibler divergence (Cover and Thomas, 1991) and it functions as a regularizer, constraining decoded representations to be biased toward their prior distribution. This is a desirable property as it entails that, for example, a category representation acquired across numerous experiences cannot be substantially altered from a single outlier exemplar. In other words, the Kullback-Leibler divergence minimizes resources spent on encoding specific exemplars by penalizing higher discrepancies between the input and the central tendency of previous inputs.

It is known that allocated cognitive resources differs between people and can even fluctuate from moment to moment. Therefore, we made use of the β-VAE, which incorporates a non-negative parameter (β) that scales the Kullback-Leibler divergence (Higgins et al., 2017). By scaling the Kullback-Leibler divergence, the bias toward the central tendency of experience can be made more or less prominent. It is conceptually related to cognitive capacity (Bates and Jacobs, 2020), given that less reliance on priors means one can efficiently encode more specific information. Specifically, autoencoders by their very nature try to reconstruct an input, which may make them susceptible to overfitting to the identity of a stimulus (Steck, 2020). In the extreme case that an autoencoder learns to memorize every training stimulus, it would resemble the famous exemplar model (Nosofsky, 1986, 1987) of categorization. However, in the case of categories with many exemplars, this becomes computationally infeasible and thus a tradeoff must be maintained between precision of memories and resource expenditure. Because the Kullback-Leibler divergence functions as a regularizer, constraining representations to resemble prior representations, the VAE additionally minimizes the resource expenditure. Thus, by scaling the Kullback-Leibler divergence, β induces more or less reliance on the prior, effectively tilting the balance of precision and complexity toward one or the other. The relationship between β-VAEs and rate distortion theory has previously been made mathematically concrete (Alemi et al., 2017a,b).

**Figure 1:**
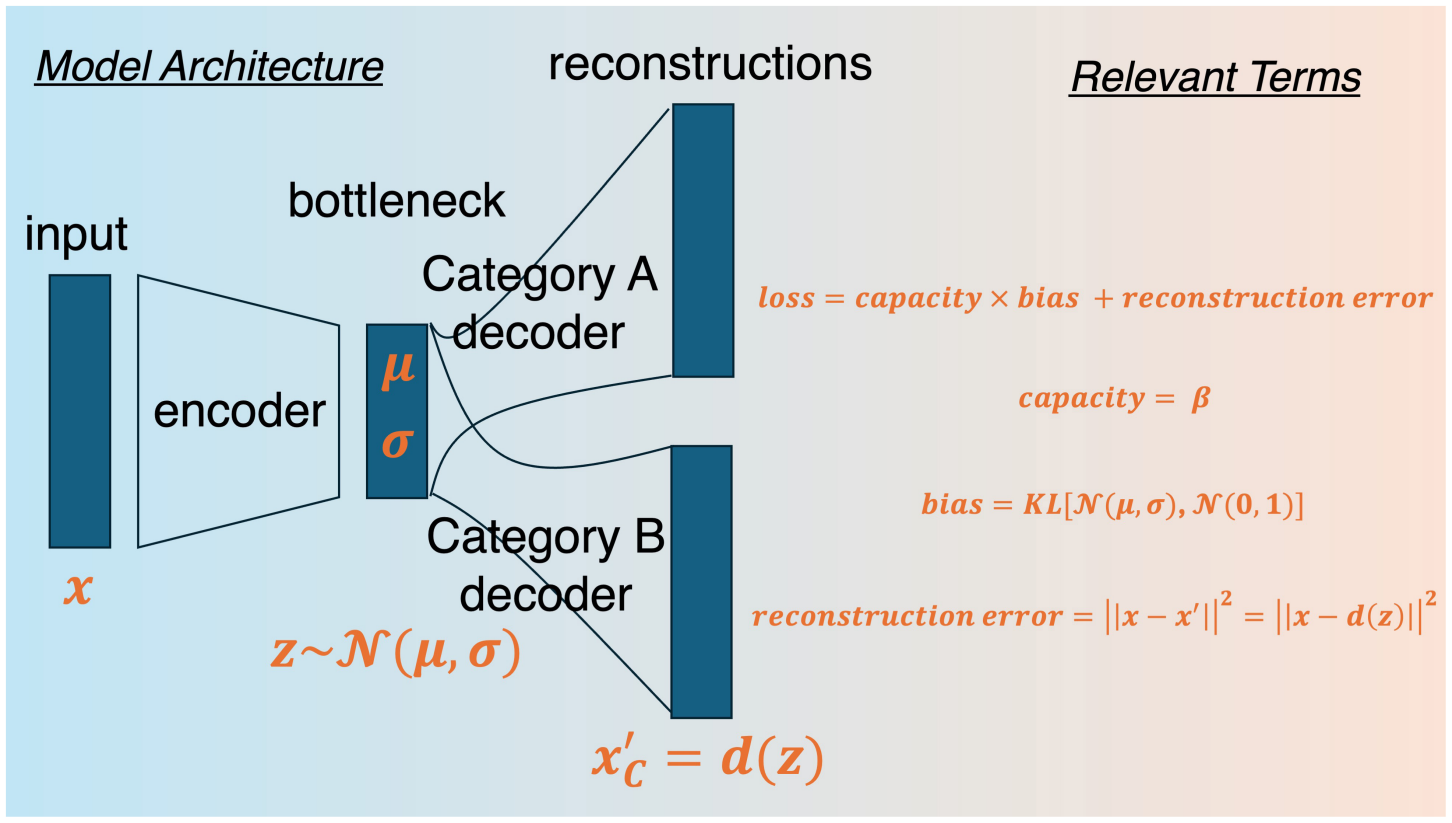
BR-DIVA model architecture. From the far left side, the model begins by taking in an input vector and projecting it onto a hidden layer (bottleneck). Then decoders for each category samples from the hidden layer space to reconstruct the input. Relevant terms reveals the loss function that gets optimized, which is a sum of reconstruction error and capacity-weighted bias. Capacity is simply a freely estimated parameter and bias is the Kullback Leibler divergence between prior and posterior distributions at the hidden layer. Reconstruction error is the squared absolute difference between input and reconstructed representations.

Finally, we make the β-VAE divergent, as in DIVA and for reasons which we expound upon next. Traditional autoencoders utilize a single decoder to decode *n*-categories, or use multiple autoencoders for each category (Oja, 1989). Such approaches to category learning do not capture differences in category learning driven by learning conditions, such as the nature and number of contrasting categories. In the former case, it is difficult to apply to supervised learning and in the latter case, this is because each category is modeled independently (Kurtz, 2007). To solve this issue, Kurtz (2007) proposed a single (shared) hidden layer of units and *n* decoders, or *category channels*, in DIVA in order to obtain reconstruction errors for each category. Comparing reconstruction errors then allows one to test the following assumption, namely that using the model's low-dimensional representation of one category to reconstruct the current stimulus is better than using the model's representation of another category to reconstruct the current stimulus. Moreover, by maintaining a shared hidden layer, DIVA and the extension proposed here are plausible models of multitask learning (Ben-David and Schuller, 2003; Caruana, 1996, 1997, 1994), which has recently been revealed to naturally facilitate generalization and abstraction (Driscoll et al., 2024; Garner and Dux, 2023; Sanh et al., 2022; Wards et al., 2023) and may be related to mixed selectivity in the brain (Jeffrey et al., 2020; Kaufman et al., 2022; Rigotti et al., 2013), including the hippocampus (Bernardi et al., 2020; Kira et al., 2023) and the prefrontal cortex (Dang et al., 2021; Parthasarathy et al., 2017), both of which are involved in concept learning. Given the shared layer, the current model claims that the bottleneck component constitutes a space of multiple psychological spaces superimposed upon each other, which is distinct from predictions made by autoencoder models with a single decoder. This means that the current model will yield different reconstructions under different learning conditions (i.e., it utilizes interdependent encoding techniques). By allocating a unique output channel for each category, divergent autoencoder architectures can model supervised learning by obtaining reconstruction errors for each category. For a schematic and relevant terms of the model proposed here see Figure 1.

To test the viability of the currently proposed model, which we call BR-DIVA (for *Boundedly-Rational-*DIVA, see below), we compare its ability to capture a classic benchmark of category learning to the original DIVA model and consider unique predictions by making DIVA variational. The aim of the current paper is to guide future research by positing a few category learning predictions that follow logically from computational principles.

### Model features

The VAE model proposed here is a neural network model with three layers composed of three, two, and six neuron-like units, respectively. The number of units per layer were selected based on the stimulus set used in the current study. Because the stimuli are three-dimensional, the input layer is composed of three units and the output layer is composed to 3 x *n-categories* units. To be comparable to DIVA, which used two hidden layer units, we fixed the number of units in the hidden layer, or bottleneck to two. More details on the stimulus set are provided below. Input and output layers are fully connected with the hidden layer (i.e., the bottleneck). These connections denote the associations between input stimuli, internal cognitive representations, and reconstructions and are learned by iterative updating of weights that scale each connection strength. Unit weights are learned via standard backpropagation (Rumelhart et al., 1986) and activations are passed through a sigmoid function. Weights are updated in proportion to the learning rate. Unit weights are initialized with random values between default values of ±0.5, which is convention for neural network research (Kolen and Pollack, 1990) and used in the paper introducing DIVA (Kurtz, 2007).

Activations spread from input to hidden layer units. The hidden layer is comprised of two neuron-like units, which is what gives it its status as a bottleneck. That is, by projecting three-dimensional inputs (see below) onto a two-dimensional space, the encoder is forced to reduce the input's dimensionality. Then the hidden layer projects to the output layer, which has dimensionality equal to the dimensionality of the input stimulus for each channel, which is why the output layer has 6 units (3 units for each category; see below for explanation of the stimuli).

To optimize model fit, a loss function gets minimized. The loss function is the sum of two terms: (1) reconstruction error, and (2) weighted Kullback-Leibler divergence. To obtain the measure of reconstruction error, squared differences between each category channel's output node activations and the input are calculated and scaled with a sensitivity parameter that controls the amount of attention paid to each feature. Summing these differences within each category channel yields a reconstruction error for each category. These measures are then added to the Kullback-Leibler divergence that itself gets scaled by the regularization parameter β. For additional details on how parameter settings relate to category learning, see (Kurtz, 2007, 2015). Here, we fix the sensitivity and learning rate parameters to 1 for brevity [as was done in the original DIVA simulations (Kurtz, 2007)]; and to elucidate the differences between DIVA and BR-DIVA models. DIVA also makes use of an attention breadth parameter that specifies how much attention is allocated to specific dimensions vs. all dimensions; however, to facilitate ease of comparison, this parameter was also fixed to 1 for both models.

To demonstrate the plausibility of the current model's ability to capture human category learning, we test its ability to simulate category learning on the seminal “Six Problems” introduced by Shepard et al. (1961).

## The Six Problems

Shepard et al. (1961) tested the difficulty of categorization judgments depending on how the same 8 stimuli were grouped. Specifically, participants were shown three-dimensional stimuli, where each dimension denotes a binary feature (e.g., color, size, and shape). These eight stimuli can be grouped into two groups in 70 different ways, but only six of these are structurally distinct. By “structurally distinct,” we mean that a grouping is not different simply by swapping out features. For example, a Type 1 grouping assigns all four stimuli with one color value (say, black) to Category A and all four stimuli with the other color value (say, white) to Category B. Grouping the stimuli using the same kind of unidimensional rule, simply for a different dimension (i.e., grouping all small stimuli into A and all large stimuli into B) is a technically unique grouping but not structurally distinct.

**Figure 2:**
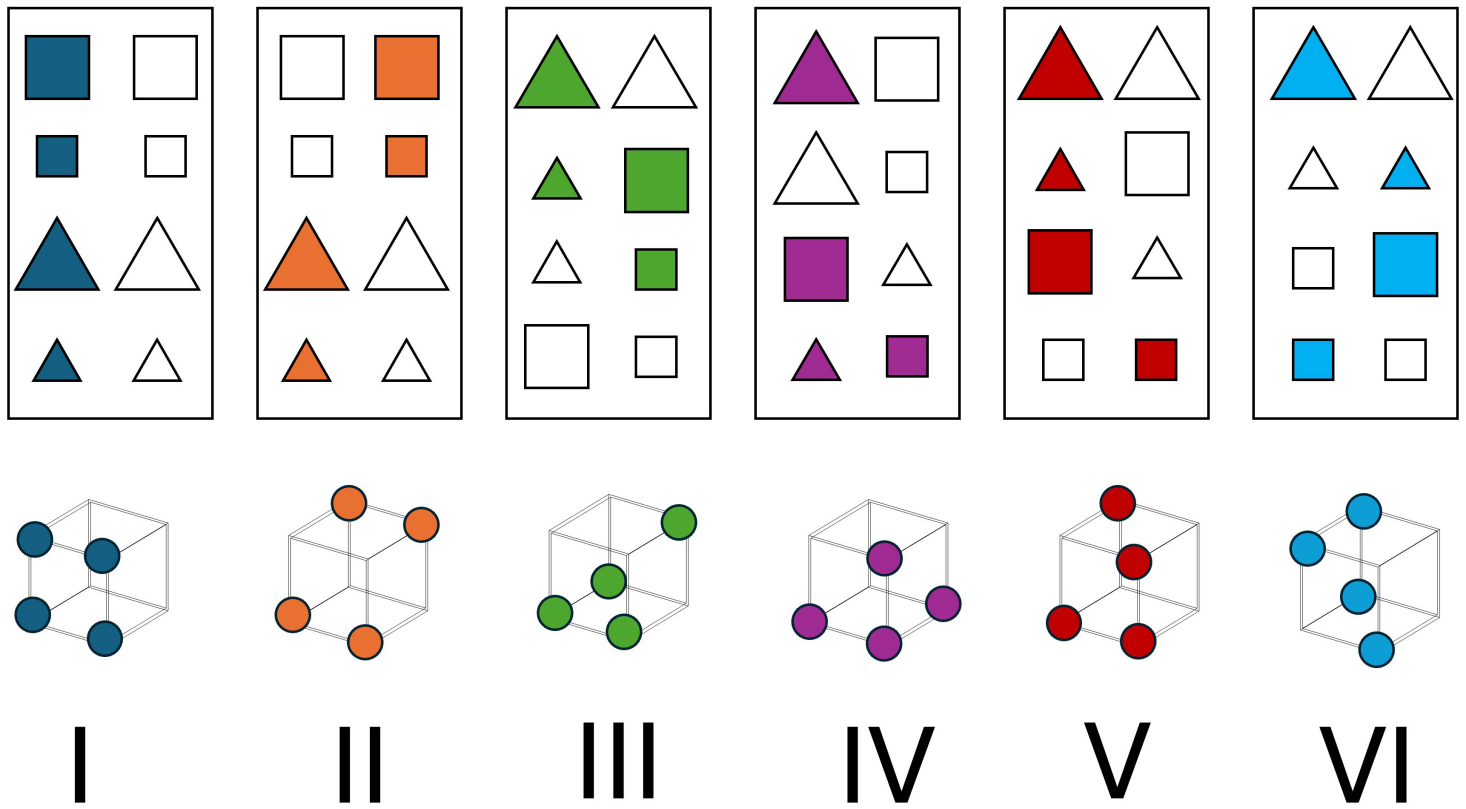
Six Problems. Every category structure implemented in the seminal paper by Shepard et al. (1961). Within each panel, each stimulus on the left belongs to one category and all the stimuli on the right belong to another category. Below the top panels is a 3-dimensional representation of the each category structure.

The six types of groupings differ in the number of dimensions one must attend to in order to achieve optimal performance (Type 1: one dimension, Type 2: two dimensions, and Types 3–6: three dimensions). Type 1 adheres to a unidimensional rule-based structure, such that all stimuli with one value on a dimension (e.g., color in Figure 2) belong to Category A while all stimuli with the other value on the same dimension belong to Category B. Type 2 is an exclusive-OR (XOR) problem, where two dimensions are relevant. In Figure 2, Category A stimuli can be white and square or orange and triangle. Types 3, 4, and 5 can all be characterized as rule-plus-exception structures, where a single dimension defines category assignments for three of the category's four stimuli and thus the fourth stimulus for each category must be memorized. Type 6 is the most difficult because it lacks any within-category similarity structure, meaning one must memorize each of the eight stimulus-response associations to perform optimally. Figure 2 shows an example for each of the six types.

The main findings (i.e., that performance follows difficulty level; Type 1 > Type 2 > Types 3–5 > Type 6) from the Six Problems introduced in Shepard et al. (1961) have been replicated many times, with larger sample sizes, diverse stimulus sets, and across species (Kurtz et al., 2013; Nosofsky et al., 1994a; Smith et al., 2004).

## Method

We simulated *n* = 100 participants that performed each of the Six Problems. The model was constructed as a stateful list processor (see Wills et al., 2017) and used the *slpDIVA* function (DIVA model) from the R package *catlearn* (Wills et al., 2017) as a starting point. The current model begins each simulation with randomly initiated weights. A binary three-dimensional input, representing one of the eight stimuli from the Six Problems (i.e., a trial), serves as the first layer and is mapped to 2 probability distributions (i.e., the bottleneck) via matrix multiplication with a set of input weights. These distributions are reparameterized via the reparameterization trick (Kingma et al., 2015). Reparameterized means of these distributions are the hidden unit activation levels which are then projected to two three-dimensional output layers via a set of output weights for each category. The output weights represent input reconstructions. A category judgment, which gets a 1 or 0 for accuracy, is whichever category has less reconstruction error. One simulation is 20 blocks of category learning, where a single block is one iteration through all eight stimuli, presented to the model in random order. We tested both the BR-DIVA and original DIVA model in order to test for any additional benefit of making DIVA variational.

We ran the above procedure for each of 50 different β values, from 0.01 to 100 in evenly spaced increments on a logarithmic scale. By fixing the parameters common to both the original DIVA model and the currently proposed BR-DIVA model, we can succinctly evaluate the contribution that bounded rationality makes to the divergent autoencoding architecture of category learning.

We conducted statistical analysis on the simulated performances from both BR-DIVA and DIVA. All analyses were done on accuracy (proportion correct), though plots show error rate (proportion incorrect) to facilitate easy comparison with previous studies studying the Six Problems. To test the extent to which BR-DIVA's category learning reflects the order of difficulty observed in the Six Problems, we ran a simple linear regression, predicting aggregated performance (overall mean accuracy) from problem type and β parameter value. We ran the same tests for performance from DIVA model (without the β parameter predictor). We ran *post-hoc* paired samples *t*-tests when necessary. To compare performance to empirical data, we obtained public datasets deposited in the R package *sixproblems*. These datasets are from Nosofsky et al. (1994a) and Lewandowsky (2011), and we will refer to these datasets as nosofsky94 and lewandowsky11 for simplicity. We briefly describe these datasets below.

After comparing overall performance, we evaluated differences in performance over time (learning curves) between BR-DIVA and DIVA. We conducted simple linear regression models predicting accuracies from block and type for both models.

Nosofsky94 is comprised of 120 participants. Each participant performed two problem types and each problem type was administered an equal number of times. Thus, there were 40 participants assigned to each problem type. The order of problem type assignment to each participant was counterbalanced. The first two blocks comprised one showing of each of the eight stimuli and all subsequent blocks comprised two showings of each of the eight stimuli. Participants continued the task until reaching a criterion of four consecutive sub-blocks of eight stimuli with perfect accuracy or for a maximum of 25 blocks.

Lewandowsky11 is comprised of 113 participants, who each did all six problem types in counterbalanced order. Each problem type was studied for a maximum of 12 blocks, where each block featured 2 showings of each of the eight stimuli. Study was terminated if accuracy was perfect for two consecutive blocks.

To compare learning curves predicted by BR-DIVA with observed data, we ran a mixed effects linear regression model using the *lmer* function from R's lmerTest package. This model predicted accuracy from problem type (3–5), block, and their interaction. We also included subject IDs and which dataset the data came from Nosofsky94 or Lewandowsky11 as random effects. For effects of problem type, Type 5 was entered into the model as the reference group. Thus, positive coefficients for Types 3 and 4 indicate higher accuracy than Type 5, and vice versa.

## Results

### Order of difficulty

**Figure 3:**
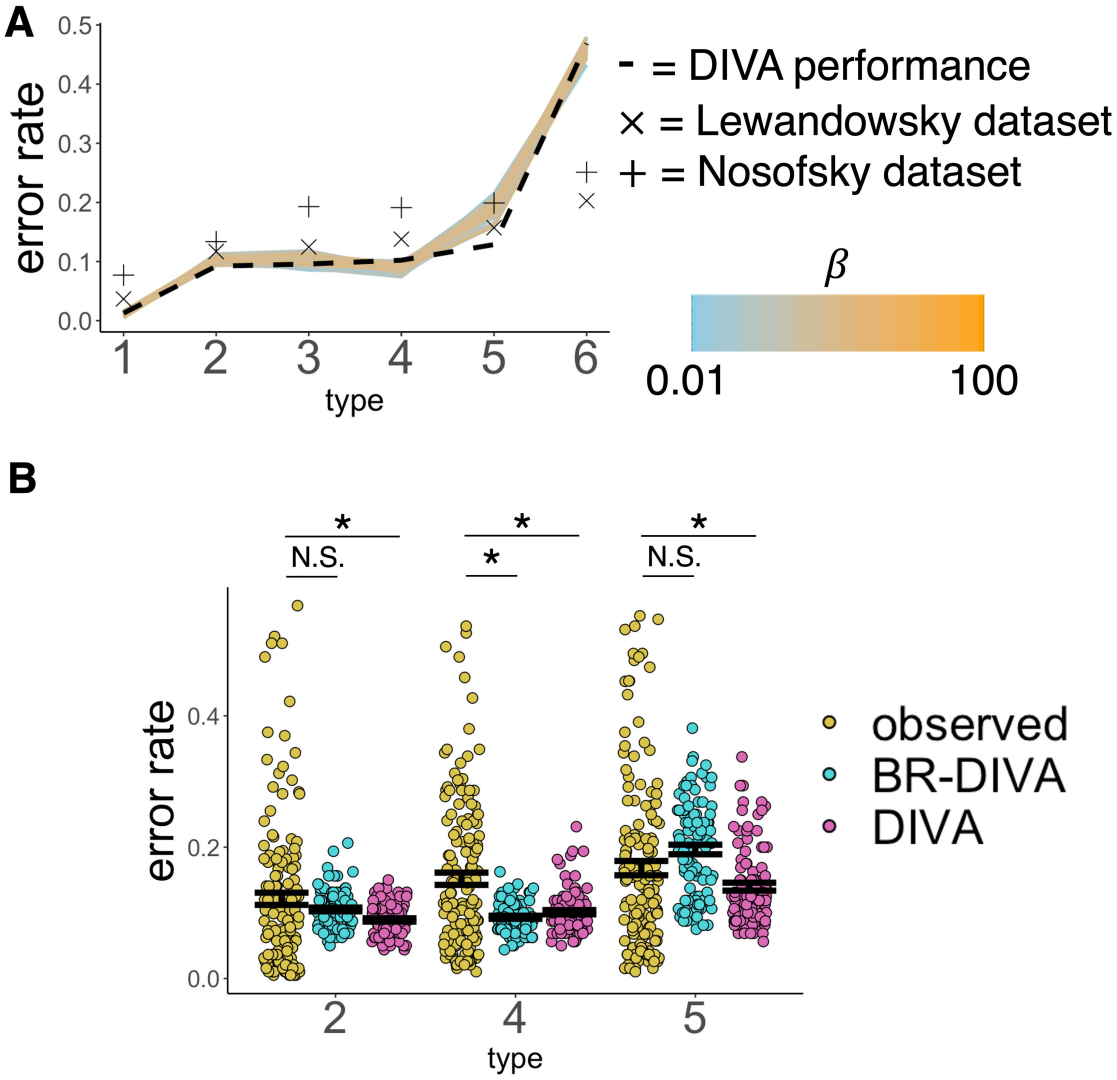
Overall model performance on the Six Problems. **(A)** X-axis denotes problem type and the y-axis denotes the overall mean performance. Colored lines are BR-DIVA predictions assuming β values ranging from 0.01 (light blue) to 100 (orange). The dashed line are predictions made by DIVA. Xs are empirically observed performances from Lewandowsky (2011) and +s are empirically observed performances from Nosofsky et al. (1994a). **(B)** Overall mean accuracy predicted by both BR-DIVA (blue) and DIVA (pink) and observed performance from participants from both Lewandowsky (2011) and Nosofsky et al. (1994a). Dots represent individual participants or simulated participants. Error bars are ±SEM. ^*^ < 0.05. NS > 0.05.

The relative ease of acquisition of category knowledge across the Six Problems introduced in Shepard et al. (1961) was tested in the boundedly rational model proposed here. We first ran a simple linear regression, predicting average proportion of correct responses (across simulated subjects and blocks) from type (1–6) and β. Please note that all β*s* with associated *p*-values below are referring to regression coefficients and not the model parameter. This reveals significant main effects of all types (β_1 − 2_ = −0.09, *p* < 0.001; β_1 − 3_ = −0.09, *p* < 0.001; β_1 − 4_ = −0.08, *p* < 0.001; β_1 − 5_ = −0.1, *p* < 0.001; β_1 − 6_ = −0.4, *p* < 0.001). Moreover, visual inspection of Figure 3A tells us that performance follows the order of difficulty typically observed. Supplementary Figure 1 additionally shows that BR-DIVA, like the original DIVA, can capture the revised ordering of the Six Problems, as elucidated in Kurtz et al. (2013). Further, the BR-DIVA model performance remains relatively stable across all tested β values, at least at the aggregated level (Figure 3A). Paired-samples *t*-tests showed that BR-DIVA predicts worse accuracy than DIVA on Type 2 [*t*_(99)_ = −3.17, *p* = 0.002] and, more prominently, Type 5 [*t*_(99)_ = −5.76, *p* < 0.001], and predicted significantly better accuracy than DIVA on Type 4 [*t*_(99)_ = 4.20, *p* < 0.001]. All other *ps* > 0.402. Overall sums of squared differences between error probabilities as observed in Nosofsky94/Lewandowsky11 and both BR-DIVA and DIVA are reported in Supplementary Table 1.

To determine whether these unique predictions made by BR-DIVA better reflect empirical performance than DIVA, we compared performance to that reported in Nosofsky et al. (1994a) and Lewandowsky (2011). We ran six two-samples *t*-tests, comparing simulated performances by BR-DIVA and DIVA on Types 2, 4, and 5 with subject averages from Nosofsky et al. and Lewandowsky on the same problems. We collapsed across both datasets, but running the analyses on each dataset separately support the same conclusions. Both models predict significantly better accuracy on Type 4 than is actually observed [BR-DIVA: *t*_(251)_ = 5.63, *p* < 0.001; DIVA: *t*_(251)_ = 4.18, *p* < 0.001]. Intriguingly, however, while DIVA predicts significantly more categorization accuracy than is actually observed for both Types 2 [*t*_(251)_ = 2.49, *p* = 0.014) and 5 [*t*_(251)_ = 2.80, *p* = 0.005], BR-DIVA's predictions statistically match observed performances [Type 2: *t*_(251)_ = 1.50, *p* = 0.135; Type 5: *t*_(251)_ = −1.38, *p* = 0.170]. Thus, at the aggregate level, BR-DIVA makes many of the same predictions as DIVA with respect to the Six Problems, as is to be expected given that BR-DIVA is a variational version of DIVA. However, BR-DIVA makes aggregate predictions for Types 2 and 5 that are statistically similar to what is empirically observed in people whereas DIVA does not (assuming all shared parameters are the same across models; Figure 3B).

### Learning curves

**Figure 4:**
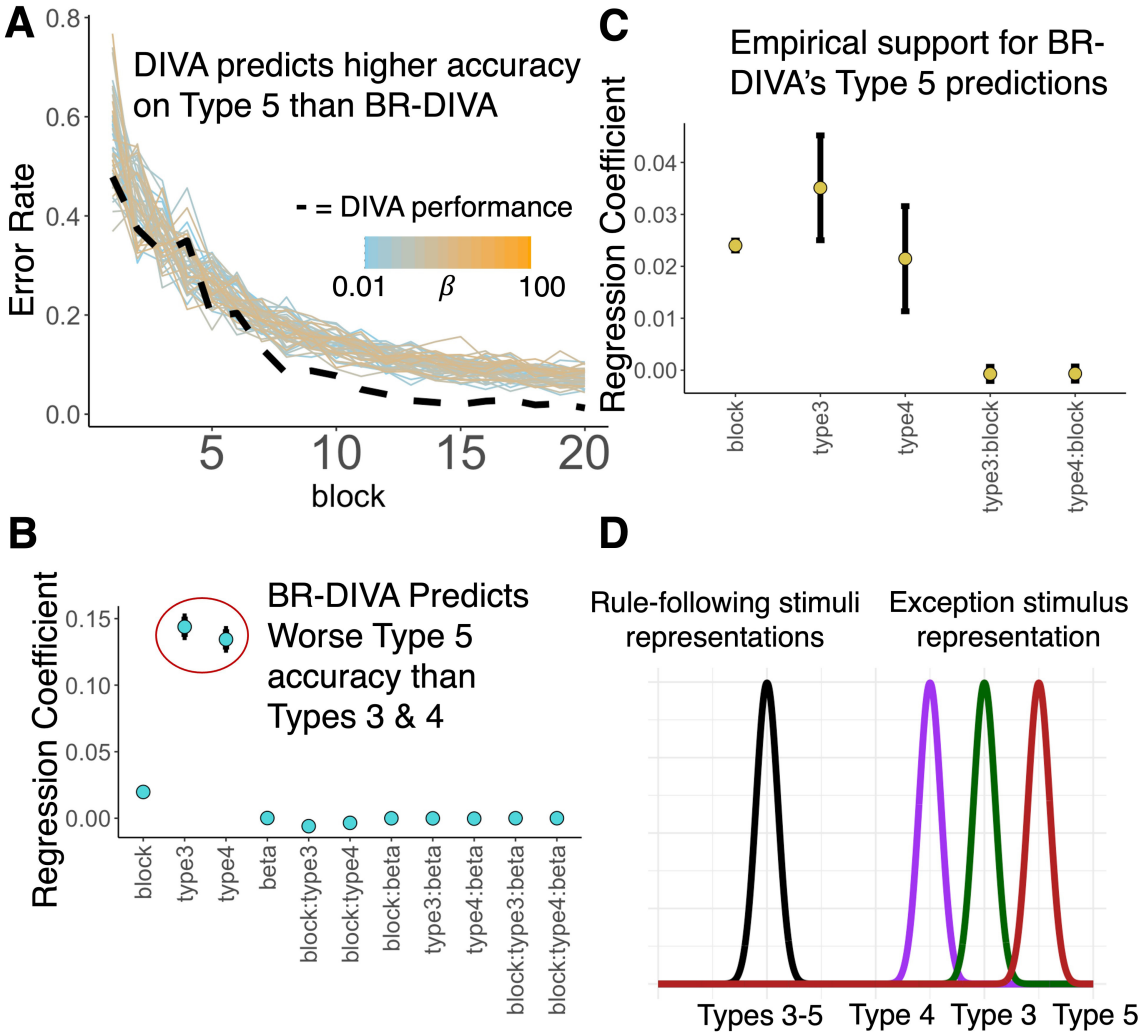
BR-DIVA and observed data suggest Type 5 is harder to learn than Types 3 and 4. **(A)** Learning curves predicted by BR-DIVA (colored lines) and DIVA (dashed line). Colored lines are BR-DIVA predictions assuming β values ranging from 0.01 (light blue) to 100 (orange). **(B)** Coefficients for predictors denoted along the x-axis from linear regression model predicting accuracy across blocks for the BR-DIVA model. Both circled coefficients are significantly different from 0 (to reiterate, the effects of problem type, Type 5 was entered into the model as the reference group). **(C)** Coefficients for predictors denoted along the x-axis from a mixed effects model predicting accuracy across blocks for data obtained from Lewandowsky (2011) and Nosofsky et al. (1994a). Regression coefficients for Type 3 and Type 4 are significantly different from zero. Error bars are ±SEM. **(D)** Conceptual schematic explaining why BR-DIVA predicts worse performance on Type 5 than Types 3 and 4 (which also explains its better Type 4 than Type 3 prediction). The black distribution on the left represents a prior distribution for a category representation represented in the bottleneck layer. This distribution is assumed to be learned by rule acquisition, as Types 3–5 all adhere to a rule-plus-exception category structure. Given that this distribution is a category representation, then learning the exception stimulus for each of these types will require this distribution to expand to incorporate the exception. As such, learning the exception stimulus should be a function of its distance from the prior distribution (in the plot, distance along the x-axis). Type 4′s exception is closest to its rule-followers, Type 3′s exception is second-closest to its rule-followers, and Type 5′s exception is furthest from its rule-followers.

To obtain a finer-grained perspective of category learning, we next looked at the learning curves for BR-DIVA. We found that BR-DIVA learns at a similar rate to DIVA for Types 1, 2, 3, and 4, and that learning is relatively stable across different values for β. For Type 5, BR-DIVA and DIVA clearly make different predictions (by the final block, BR-DIVA's best performance, across β*s*, was 96% accuracy, which DIVA surpasses on the 13th block; Figure 4A). Moreover, DIVA's learning curve for Type 6 appears to fluctuate more erratically than BR-DIVA's performance. To follow-up on these observations, we ran two linear regression models, predicting model accuracy on either Type 5 or Type 6 from block (1–20), model (BR-DIVA, DIVA), and their interaction. Please note that all β*s* with associated *p*-values below are referring to regression coefficients and not the model parameter.

The regression model predicting Type 5 performance showed only a main effect of block (β = 0.02, *p* < 0.001; all other *ps* > 0.177), meaning both models successfully learned the category structure over time. Similarly, the regression model predicting Type 6 performance showed a main effect of block (β = 0.02, *p* < 0.001), but also a marginal effect of model [β(*BRDIVA*−*DIVA*) = 0.02, *p* = 0.064]. Supplementary Figure 2 shows learning curve predictions for BR-DIVA at all tested βs and DIVA.

Given the consistent differences between Type 5 performance between BR-DIVA and DIVA (Figures 3B, 4A), we ran an additional test to try and formulate a specific prediction that could guide future empirical research. Given that many studies on the Six Problems focus on Types 1, 2, 4, and 6 only (Kurtz et al., 2013; Love, 2002; Love and Markman, 2003; Minda et al., 2008; Rabi and Minda, 2016; Rehder and Hoffman, 2005a), likely because Types 3, 4, and 5 tend to be lumped together due to similar performance on these problems (Nosofsky et al., 1994a; Shepard et al., 1961), it is perhaps notable that BR-DIVA predicted worse performance on Type 5 than DIVA and that BR-DIVA captured the empirical data for this category structure better. Therefore, we ran an additional linear regression model, predicting BR-DIVA accuracies from Types 3, 4, and 5 from block (1–20), Type (3–5), β*s*, and all interactions. Indeed, this model showed that Type 5 accuracy was significantly lower than both Types 3 (β_3 − 5_ = 0.14, *p* < 0.001) and 4 (β_4 − 5_ = 0.14, *p* < 0.001). This model also revealed significant Type 3 x block (β_3, *block*−5, *block*_ = −0.006, *p* < 0.001) and Type 4 x block (β_3, *block*−5, *block*_ = −0.003, *p* < 0.001) interactions, such that learning curves were steeper for Type 5. See Figure 4B for all model predictor effects.

To test the extent to which these unique predictions made by BR-DIVA are reflected in the real world, we ran a linear mixed effects model, predicting correct responses by participants from two previously collected datasets (Lewandowsky, 2011; Nosofsky et al., 1994a) from type (3–5), block, and their interaction. We also included subject IDs and which dataset the data came from as random effects. As was expected, there was a main effect of block (β_*block*_ = 0.02, *p* < 0.001); however, consistent with the predictions made by BR-DIVA, there were also main effects of Type 3 (β_3 − 5_ = 0.04, *p* < 0.001) and Type 4 (β_4 − 5_ = 0.02, *p* = 0.034). Interactions between block and Types 3 and 4 were not statistically significant (both |βs| < 0.001, both *ps* > 0.604). See Figure 4C for all model predictor effects. Figure 4D shows a schematic meant to visualize a plausible explanation for these results, which is further expounded upon in the discussion. Additionally, Supplementary Figure 3 shows the low-dimensional representations of each category for BR-DIVA, as well as inter-item distances in the low-dimensional space, which reveals that BR-DIVA represents Type 5 exception stimuli as further from rule-following stimuli than for Types 3 and 4 exception stimuli. Supplementary Figure 4 provides further evidence for this notion that Type 5 difficulty is a function of its inter-item distances by visualizing error rates across blocks split into rule-following and exception stimuli. Whereas, for Types 3 and 4 exception stimuli are learned at a pace similar to their rule-following stimuli, Type 5 shows that exception stimuli error rates remain higher than rule-following error rates until roughly the 15th block. Notably, however, this interpretation is incomplete as Supplementary Figure 3 shows that low dimensional representations of Type 4 exception stimuli are further from rule-following stimuli than Type 3′s exception stimuli.

## Discussion

In this brief report, we simulated performance on the canonical Six Problems known to elucidate general category learning behavior (Shepard et al., 1961) using an autoencoder model that applies principles of efficient coding (Barlow, 2013) to encode information in a boundedly rational manner. We showed that this model—BR-DIVA—captures the classical order of difficulty observed on the Six Problems (Nosofsky et al., 1994a; Shepard et al., 1961). Beyond these findings, the boundedly rational model proposed here predicted lower accuracy on Type 5 than what is predicted by the autoencoding model it is based on. Importantly, we found that this unique prediction is more aligned with empirical data than the base model. We discuss and speculate on this finding next.

### Type 5 is more difficult than Types 3 and 4

The classical Six Problems of category learning introduced in Shepard et al. (1961) produced substantial excitement about Types 1, 2, 4, and sometimes 6. Many studies that use the Six Problems only focus on this subset (Kurtz et al., 2013; Love, 2002; Minda et al., 2008; Rabi and Minda, 2016; Rehder and Hoffman, 2005b). Since the findings from Shepard and colleagues, there has been a tendency to lump performance on Types 3–5 together, as if they were the same category structures. Indeed, they do all adhere to a rule-plus-exception design (Nosofsky et al., 1994b); however, it is perhaps notable that the boundedly rational model put forth in the current paper consistently predicted worse performance on Type 5 than Types 3 and 4. This prediction did not reach statistical significance in the model on which the boundedly rational model is based on (i.e., DIVA). When comparing boundedly-rational-DIVA and DIVA to empirically observed performance differences between Type 5 and Types 3 and 4, we found that the data is more consistent with the boundedly-rational-DIVA's predictions.

One possible explanation for this discrepancy is in terms of information gain, which expresses the amount of information gained about a signal by observing another variable (Mathy, 2010). For example, by learning the weather one is likely better able to gauge what clothes a random person will be wearing. Thus, knowing the weather reduces one's uncertainty about what clothes people will be wearing. In terms of the Six Problems, information gain is relevant because it denotes the amount of information a given stimulus supplies about the categories. This notion is particularly important for rule-plus-exception category structures because it is assumed that people will learn the unidimensional rule first (Figure 4D, black distribution), in which case learning of the exception stimulus (Figure 4D, colored distributions) is a function of how distinct it is from the rule-following stimuli (Figure 4D, distance between black and colored distributions). In other words, learning a rule first to categorize stimuli will induce a bias toward the rule-following stimuli. As such, the more distinct (i.e., the more informative or the further from the bias) the exception stimulus is, the harder it will be to learn it. Consistent with this interpretation, the exception stimulus in Type 5 has a larger average distance from Type 5′s rule-following stimuli than Types 3 or 4. This within-category distance measure is proportional to a commonly used metric known as *structure ratios* (Conaway and Kurtz, 2017). This interpretation is also in line with Nosofsky et al. (1994b)'s RULEX model, which suggests that people test simple rules first and gradually hypothesize more complex rules if the simpler ones fail. In Supplementary Figure 3, the hidden unit activations for each of the eight stimuli in Type 5 from a representative simulation are plotted and visualized based on both category and whether the stimulus adhered to a unidimensional rule or not. Interestingly, this figure shows that exception stimuli are represented as further from rule-following stimuli within the same category (e.g., compare inter-item distances between red triangles and red circle, and between blue triangles and blue circle). Supplementary Figure 3 also shows that these distances are significantly greater than rule-to-exception stimulus distances for Types 3 and 4. Thus, the low-dimensional representations of stimuli are consistent with the interpretation visualized in Figure 4D. Together, this highlights the importance of priors during the process of learning categories and that, at least some, category structures' difficulty is a function of balancing representational precision with complexity.

### Limitations

The current work is not meant to encompass all categorization phenomena. Indeed, the current work only tested one category learning paradigm (i.e., classification), comprised of relatively simple stimuli. The simplicity of the stimuli actually limits the amount of dimensionality reduction that could be performed by BR-DIVA in the current work, given that stimuli were three-dimensional and the bottleneck layer was two-dimensional. This could also be why there was no difference across simulations with different β*s*. Future work will need to test for BR-DIVA's applicability to higher dimensional, naturalistic, and continuous stimuli, in addition to other paradigms, such as inference training and function learning. The current work was meant to take the first steps toward more broader applications, and thus, we generated BR-DIVA predictions and compared them with empirical data. Future studies will need to pit BR-DIVA against leading computational models of categorization such as SUSTAIN (Love et al., 2004), and prototype and exemplar models (Minda and Smith, 2002; Nosofsky, 1986, 1987, 1992; Smith and Minda, 2000, 2002). Moreover, while we did observe differences between Type 5 and Types 3 and 4 in empirical data, the analysis revealing this difference was targeted by using a subset of the overall dataset (only Types 3–5). As such, and in combination with many previous studies showing minimal performance discrepancies between these category structures, it is likely that this effect is quite subtle and future studies will need to test this prediction explicitly before any conclusive interpretations can be made.

## Data Availability

The original contributions presented in the study are included in the article/Supplementary material, further inquiries can be directed to the corresponding author.
