# Supplementary material for: A boundedly rational model for category learning
Source: Front Psychol. 2024 Dec 9;15:1477514. doi: 10.3389/fpsyg.2024.1477514 (PMC11663663; doi:10.3389/fpsyg.2024.1477514)
Supplement: Supplementary file 1 [file Table_1.DOCX]

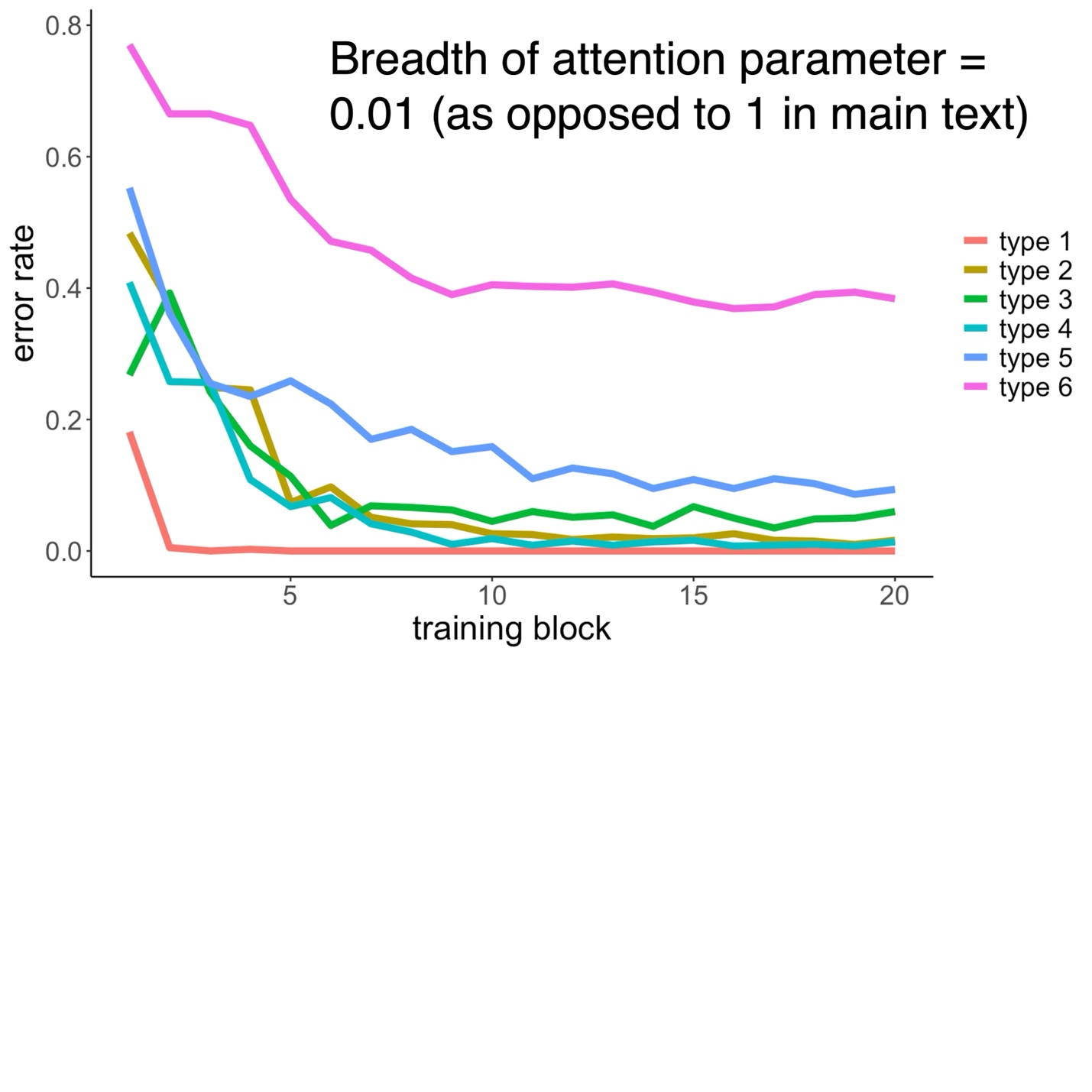


**Supplementary Figure 1.** BR-DIVA predicts revised ordering of the Six Problems. The revised ordering of the Six Problems revealed that Type 2 was as difficult to learn for people as Type 4 when explicit instruction to try to find a rule for category membership was not supplied in instructions. As in the original DIVA model, reducing the attentional focus parameter increases attentional breadth, which is likely how attention is at least initially distributed when learning the Type 2 problem without the instruction to search for a rule (which would induce attentional focus on specific stimulus features). X-axis denotes the training block, y-axis denotes error rate or proportion of incorrect responses made by BR-DIVA, and color denotes the type. $\beta$ was fixed at 1.


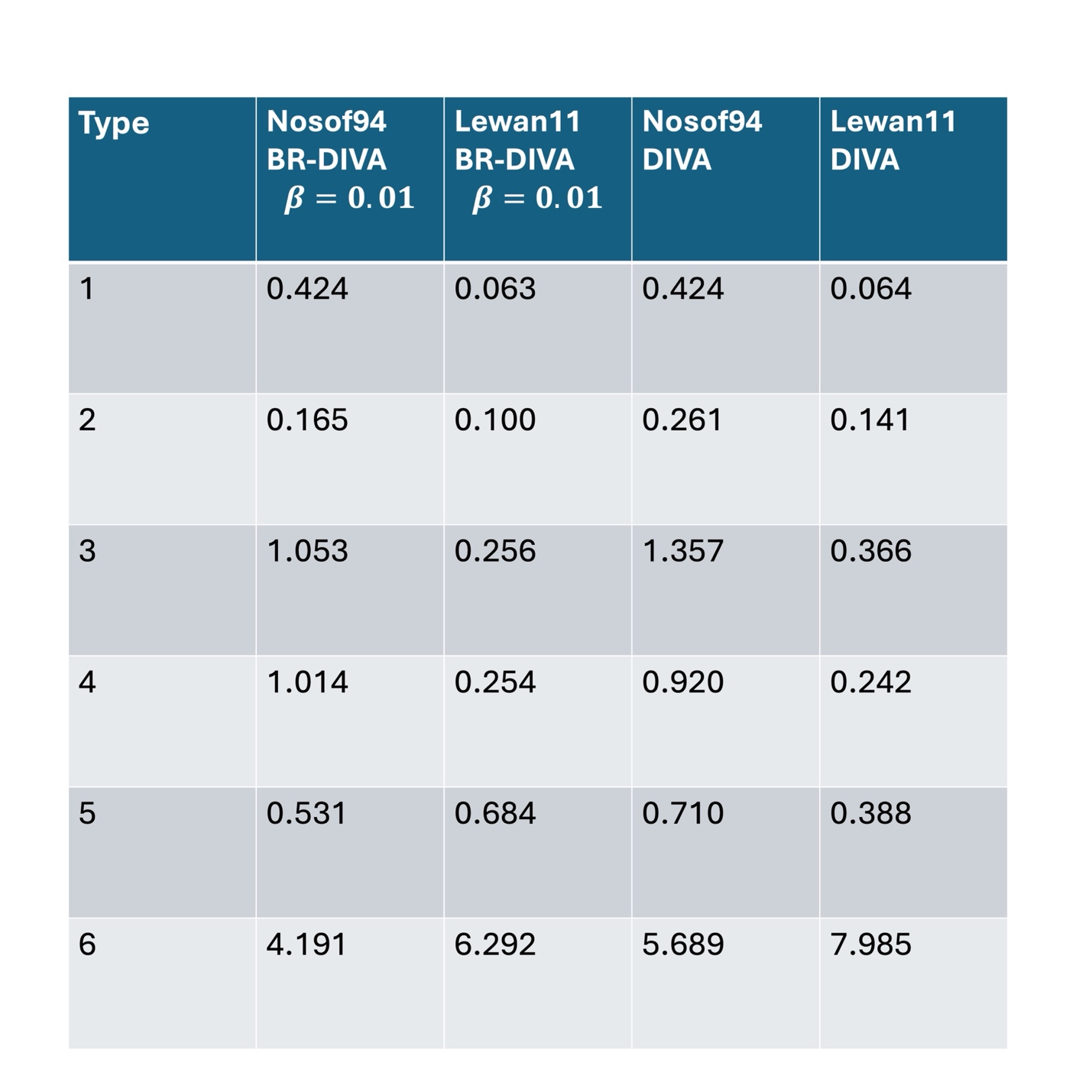


**Supplementary Table 1. Sums of squared differences between error rates of model (BR-DIVA/DIVA) and observed error rates (in nosofsky94/lewandowsky11).**


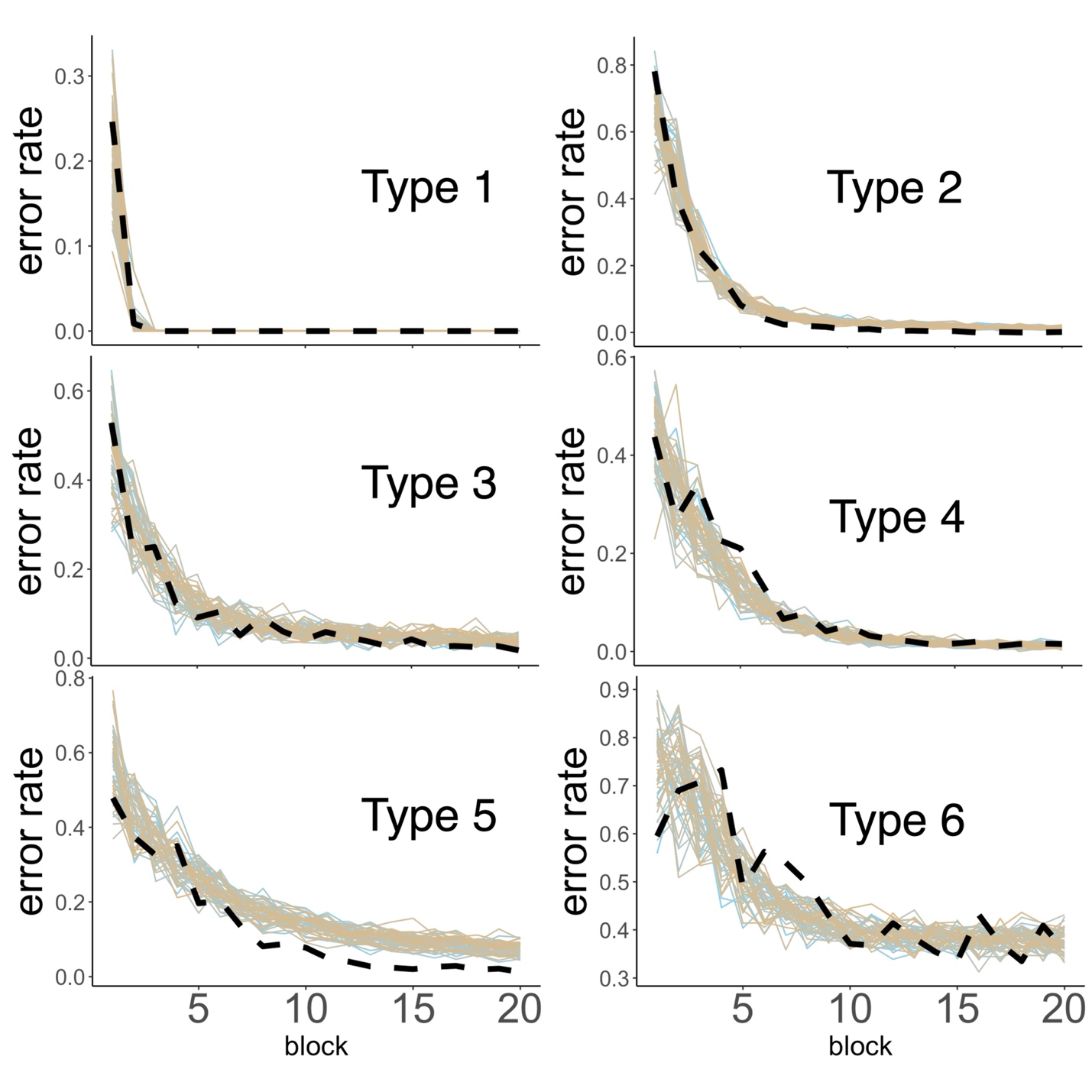


**Supplementary Figure 2.** Error rates across blocks for all Six Problems. Colors denote predicted error rates from BR-DIVA simulations and the dashed black line denotes predicted error rates from DIVA simulations.


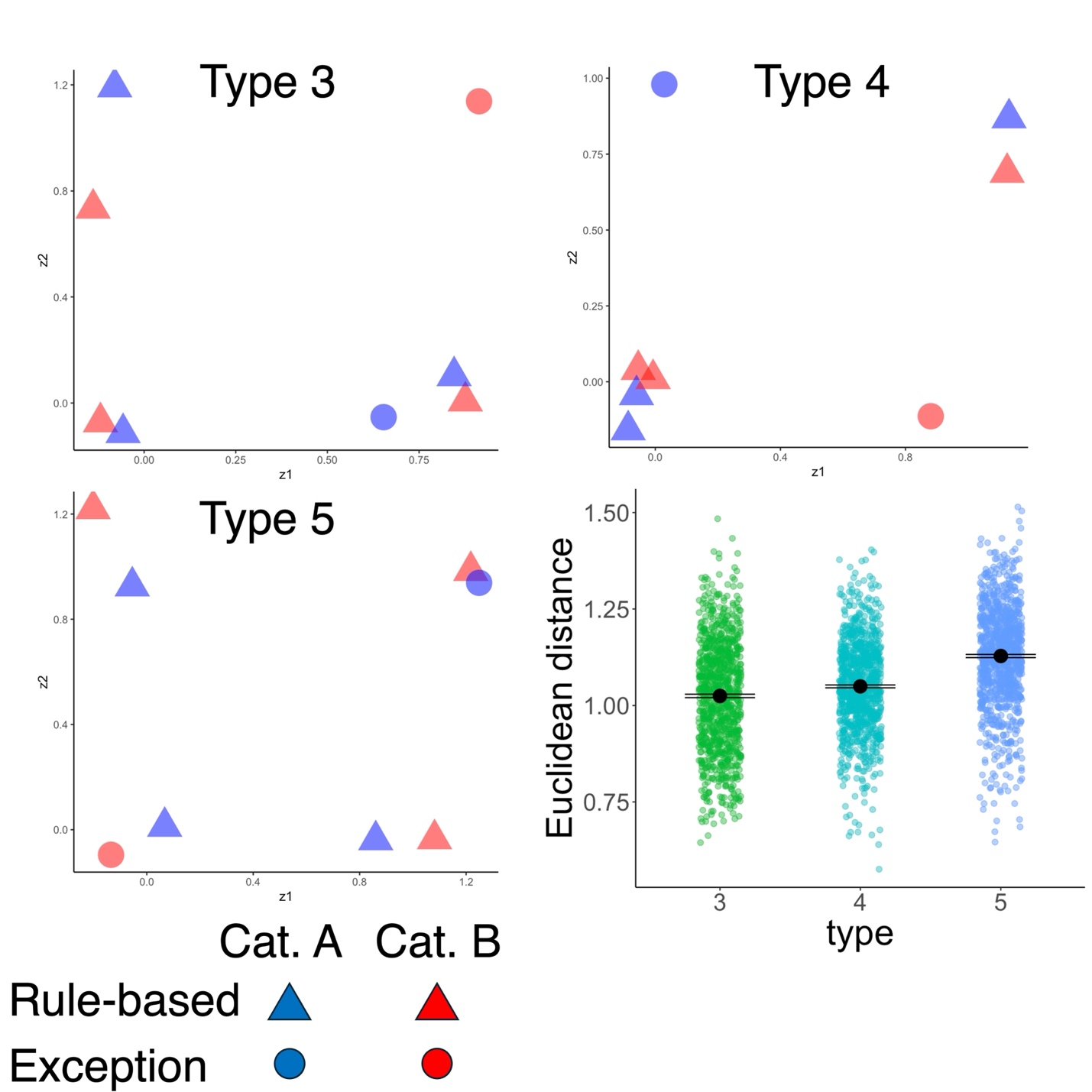


**Supplementary Figure 3.** Representative hidden unit activations from bottleneck layer for each of the 8 stimuli during the final block of training on Types 3 (**A**), 4 (**B**), and 5 (**C**), as predicted by the BR-DIVA model. Points are representative unit values from the bottleneck layer of the model. X-axis denotes one hidden unit activation level and y-axis denotes the second hidden unit activation level. Color denotes correct category membership. Shape denotes whether the stimulus was rule-following or an exception to its respective category. **D.** Euclidean distances between rule-following stimuli and exception stimuli on the last block of training. Points are averaged first within, then across categories. That is, first the mean Euclidean distance was calculated between the three rule-following stimuli and the exception stimulus within each category. Then these two measures were averaged. This was repeated 1000 times, resulting in the distribution of rule-exception distances in **D**. Type 5 distances are significantly greater than Types 3 and 4 distances.


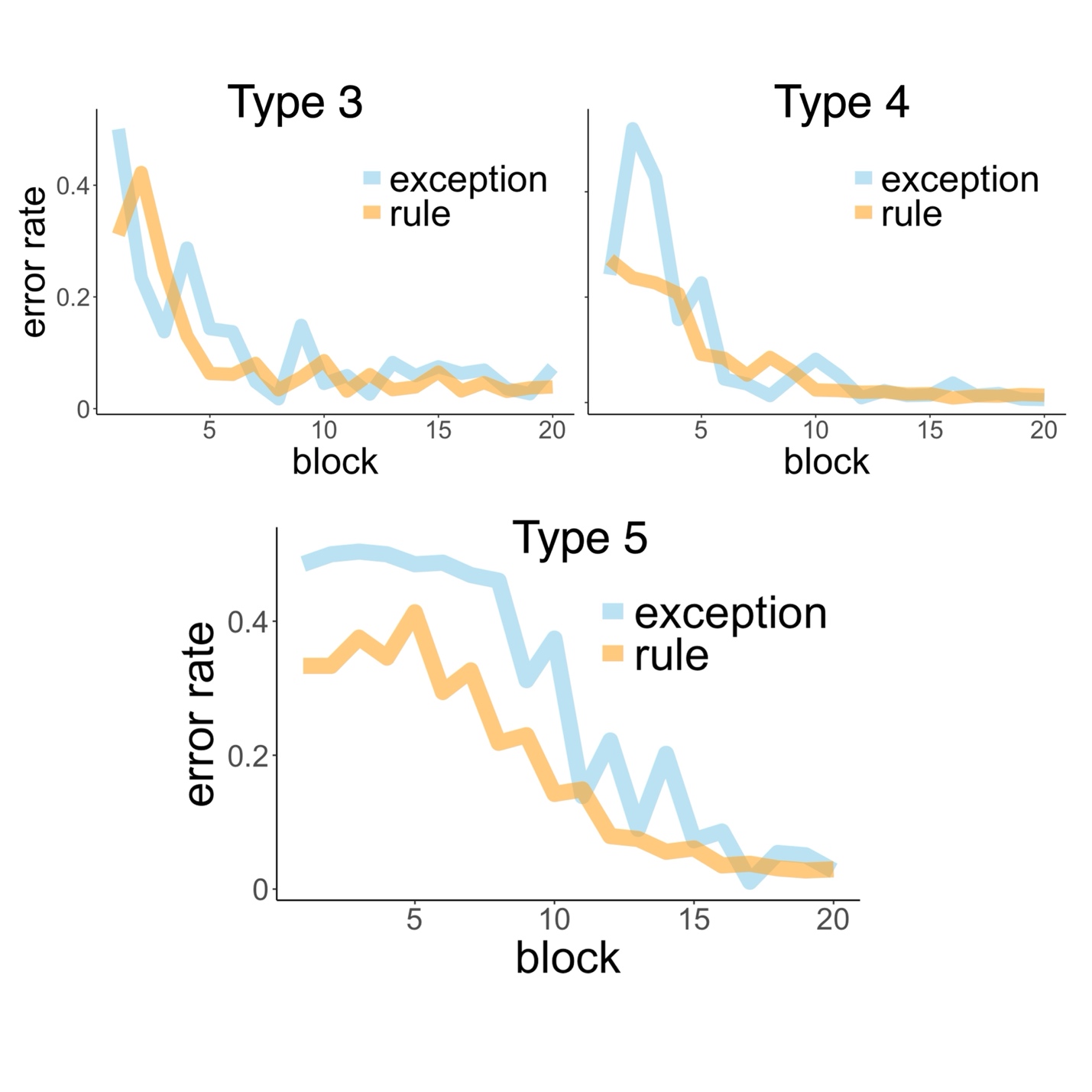


**Supplementary Figure 4**. Learning curves for Types 3-5 split into performance on rule-following and exception stimuli. Y-axis is the error rate, or proportion of incorrect responses predicted by BR-DIVA, averaged across 1000 simulations. X-axis denotes training block and color denotes whether the error rate is for rule-following or exception stimuli.
